# Mechanics of Mindfulness: Investigating Metacognitive Beliefs as a Pathway of Effect on Anxiety and Depression

**DOI:** 10.3390/ejihpe15060109

**Published:** 2025-06-12

**Authors:** Corey Jackson, Christian M. Jones

**Affiliations:** Faculty of Arts, Business and Law, University of the Sunshine Coast, Sippy Downs, QLD 4557, Australia; cmjones@usc.edu.au

**Keywords:** mindfulness, S-REF, rumination, mind wandering, worry, anxiety, depression, metacognitive beliefs, dereification

## Abstract

This study aimed to address the dearth of literature on mechanisms of effect of mindfulness-based interventions by investigating metacognitive beliefs as a potential mechanism of symptomology-reduction effects. The Cognitive Attentional Syndrome (CAS) component of the Self-Regulatory Executive Function (S-REF) model was augmented to include subtypes of mind wandering and rumination. One hundred and seventy-eight participants sourced from professional networks (*M_age_* = 53.13; *SD* = 11.80) completed an online questionnaire measuring dispositional mindfulness, metacognitive beliefs, rumination, mind wandering, worry, anxiety and depression. Effects of meditation frequency on these variables were examined, as were the relationships between them. Dispositional mindfulness was significantly negatively correlated with metacognitive beliefs, which were positively correlated with worry, mind wandering and rumination, all of which were positively correlated with symptomology. Significant correlations were stronger for spontaneous mind wandering and brooding rumination than their counterparts. Those reporting a daily meditation practice scored significantly higher on three of the five facets of mindfulness and significantly lower on anxiety and depression symptomology and several CAS elements than those who rarely meditated. Changes in metacognitive beliefs are a potential pathway for MBI-driven reductions in anxiety and depression symptomology. Increases in dispositional mindfulness through MBIs are likely to reduce metacognitive beliefs, which reduce maladaptive processes of the CAS, flowing on to reductions in symptomology. A daily meditation practice appears to increase the efficacy of this mechanism. Subtypes of mind wandering and rumination differ in their contribution to this pathway, perhaps more accurately represented as extremes on their respective continua rather than the current categorical model of typologies measured independently.

## 1. Introduction

Increases in mindfulness are significantly associated with reductions in perceived stress (Van Dam et al., 2014), increases in positive affect and decreases in negative affect (Garland et al., 2015; Garland et al., 2017; Kemeny et al., 2012), and reductions in symptomology of anxiety and depression in clinical (Costa & Barnhofer, 2016; Garland et al., 2017), peri clinical (Garland et al., 2015; Geschwind et al., 2011) and sub-clinical populations (Fumero et al., 2020; Gallegos et al., 2013; O’Mahony et al., 2017; Rodriguez Vega et al., 2014; Van Dam et al., 2014; Zeidan et al., 2010). Mindfulness training has been shown to benefit populations at risk of depression or relapse (Geschwind et al., 2011; Sverre et al., 2023), can be effective in recovery and relapse prevention of substance abuse (Bowen et al., 2014), and has been associated with significantly reduced symptomology of Attention Deficit Hyperactivity Disorder (ADHD) (Mitchell et al., 2017; Poissant et al., 2020) and obsessive–compulsive disorder (OCD) (Mathur et al., 2021; Rupp et al., 2019).

Much of this research has been carried out using multi-week mindfulness-based interventions (MBI), many of which include mindfulness training components derivative of one or more contemplative traditions (Chiesa, 2013; Chiesa & Malinowski, 2011; Creswell, 2017; Kabat-Zinn, 2011). Despite these encouraging results, the mechanisms behind MBI-induced effects of reduced symptomology remain opaque and under-researched (Condon, 2019; Dimidjian & Segal, 2015; Creswell, 2017), while a lack of standardisation of definitions and pedagogy makes cross-study comparisons difficult (Creswell, 2017; Van Dam et al., 2018).

Attempts at standardisation of definitions have been made (Bishop et al., 2004), but obstacles remain. For example, a commonly used definition in the literature taken from Mindfulness-Based Stress Reduction (MBSR); “paying attention in a particular way: on purpose, in the present moment, and non-judgmentally” (Kabat-Zinn, 2011, p. 91) is not congruent with some Buddhist definitions (Dreyfus, 2011). As an exploratory study on mechanisms, this research does not focus on any specific pedagogy or terminology; however, we acknowledge this important issue in the field.

### 1.1. Background

The current dearth of understanding of the mechanics behind these effects makes the development and refinement of more efficacious MBIs somewhat reliant on guesswork and intuition. Given the apparent transdiagnostic nature of MBI effects, this research investigated the inter-relationships of mindfulness with metacognitive beliefs and other elements of the Self-Regulatory Executive Function (S-REF) model of emotional disorder (Wells & Matthews, 1996, 2015).

#### 1.1.1. Self-Regulatory Executive Function (S-REF) Model

The Self-Regulatory Executive Function (S-REF) model of emotional disorder (Wells & Matthews, 1996, 2015) is a well-established transdiagnostic model (Capobianco et al., 2018; Nordahl et al., 2019). First developed in relation to general anxiety disorder (GAD) (Wells, 2002) (for a history of this approach, see Strand et al., 2024), this model has also been linked to social anxiety disorder (Nordahl & Wells, 2019), depression (Goring & Papageorgiou, 2008; Yılmaz et al., 2011), post-traumatic stress disorder (Cook et al., 2015), smoking dependence (Spada et al., 2007), procrastination (Spada et al., 2006), pain catastrophising (Schütze, 2016) and problem drinking (Spada & Wells, 2006).

This model posits two types of worry: type 1 worry is concerned with “non-cognitive” events, and type 2 worry (also known as meta-worry) about cognitive events, sometimes explained as “worry about worry” (Wells, 2005b, p. 108). Type 2 worry is driven by erroneous metacognitive beliefs about cognition, which initiate maladaptive cognitive styles and strategies known as the cognitive attentional syndrome (CAS), through which negative affect and emotions are intensified and maintained and can lead to psychopathology (Wells, 2009).

In this model, the CAS is driven by maladaptive metacognitive beliefs, which are positively associated with the CAS. These beliefs are divided into five subcategories: (1) (lack of) cognitive confidence, (2) positive beliefs about worry, (3) cognitive self-consciousness, (4) negative beliefs about uncontrollability and danger, and (5) need to control thoughts.

Metacognitive therapy (MCT; Wells, 2010) seeks to correct and reduce these mistaken beliefs, leaving the individual with appropriate, normal worry responses to stimuli and has been shown to out-perform symptom-specific cognitive behavioural therapy (CBT) (S. U. Johnson & Hoffart, 2018; Nordahl et al., 2018).

While MCT does not include formal meditation training, an important component of the therapy is known as “detached mindfulness” (Wells, 2005a), the purpose of which is to increase awareness of cognitive events without suppressing, reacting or evaluating them. This reduced reactivity is a goal of other MBIs comprising more formal meditation practice (Kabat-Zinn, 2011), which raises the possibility that more formal training may also reduce metacognitive beliefs. To the best of our knowledge, the effects of this more formal training on the S-REF pathway to symptomology are currently unexplored.

#### 1.1.2. Rumination

Rumination is an important component of the CAS (Nordahl & Wells, 2019) and prevalent in much of the literature on transdiagnostic processes (Barrio-Martínez et al., 2023; Heeren & Philippot, 2011; Hernández-Posadas et al., 2024). The Response Styles Theory (RST) of Nolen-Hoeksema et al. (2008) defines rumination as “a mode of responding to distress that involves repetitively and passively focusing on symptoms of distress and the possible causes and consequences of these symptoms” (p. 400). It is considered one of the most consistent predictors of negative mental health outcomes (Zawadzki, 2015) and has been consistently linked to both anxiety and depression (Nolen-Hoeksema et al., 2008; Nordahl et al., 2019; Nordahl & Wells, 2019; Wells, 2009; Yapan et al., 2022).

Rumination has been reliably classified into reflective and brooding subtypes (Treynor et al., 2003), which differ in how they mediate both the onset and maintenance of depression (Schoofs et al., 2010; Treynor et al., 2003). Reflective rumination is a type of mental problem-solving to regulate negative mood and affect and is considered more adaptive than the brooding subtype (Bernblum & Mor, 2010; Eisma et al., 2015), which is a passive comparison of an individual’s current state with an unachieved desirable outcome (Schoofs et al., 2010; Treynor et al., 2003). Although reflective and brooding rumination vary in the way they relate to anxiety and depression symptomology (Heeren & Philippot, 2011; Tahtinen et al., 2019; Wang et al., 2021) and mindfulness (Josefsson et al., 2017; Kumar et al., 2008), researchers consistently report rumination as a global scale, forgoing the nuance of these subtypes (Olatunji et al., 2013).

This variance in the relationships of these rumination subtypes with other psychometric measures suggests that the manner of attending to the content of rumination episodes may play a more important role in emotional dysfunction than the content of those cognitions (Nolen-Hoeksema, 1991; Teasdale, 1988) which shows some similarity to the metacognitive model of the S-REF, where metacognitive beliefs about cognitions can be more influential than cognitions themselves. However, in its current iteration, the S-REF model does not incorporate these subtypes, instead utilising a global conceptualisation of rumination as a maladaptive cognitive strategy that is reduced by correcting erroneous metacognitive beliefs (Kowalski & Dragan, 2019; Nordahl & Wells, 2019; Wells, 2019). To the best of our knowledge, this is the first investigation into whether or how metacognitive beliefs might influence a predisposition toward one subtype of rumination or the other and how dispositional mindfulness might influence this.

#### 1.1.3. Mind-Wandering

Mind wandering is not included as a component of the CAS in the S-REF model of Wells (2019); however, it was included in this research due to recent suggestions that rumination could be considered a class of mind wandering (Fell et al., 2023) and because significant links with executive control (Seli et al., 2015b) have led some researchers to characterise mind wandering as diametrically opposed to mindfulness (Mrazek et al., 2012). However, a lack of standardised definitions of both mindfulness and mind wandering makes the veracity of this claim dependent on individual researchers’ viewpoints (Somaraju et al., 2023).

Mind wandering has shown significant positive correlations with stress and negative affect (Franklin et al., 2013; Poerio et al., 2013; Smallwood et al., 2009; Stawarczyk et al., 2013), depression (Smallwood & O’Connor, 2011), and rumination and anxiety (Fell et al., 2023). It has been linked to risks of car accidents (Yanko & Spalek, 2013), poor memory retention (Chambers et al., 2008), symptomology and negative affect associated with ADHD (Jonkman et al., 2017; Seli et al., 2015b) and obsessive–compulsive disorder (OCD) (Sánchez Escamilla et al., 2024). Mind wandering has also been found to have a significant positive correlation with adaptive traits and outcomes such as creativity (Abd-Eldayem & Shaheen, 2021; Baird et al., 2012) and efficacy in planning future events (Smallwood & Andrews-Hanna, 2013).

Disagreements such as whether a predominately future focus of mind wandering differentiates it from rumination (Mooneyham & Schooler, 2013; Poerio et al., 2013; Ruby et al., 2013; Spronken et al., 2016) exist within the literature, yet despite these, most mind wandering theories (see Banks et al., 2016) treat it as a binary phenomenon in which one is either on task or not, with off-task considered unintentional (Seli et al., 2016). These assumptions have been called into question by Seli et al. (2015a), who reliably dissociated mind wandering into two subtypes by way of intentionality. These are a deliberate (MWD) subtype in which one deliberately attends to the content of a mind-wandering episode, and a spontaneous (MWS) subtype in which one is attending to the mind-wandering content is non-volitional.

Spontaneous mind wandering (MWS) has consistently shown a stronger negative correlation to mindfulness measures than its deliberate (MWD) counterpart (Carciofo & Jiang, 2021; Carriere et al., 2013; Cásedas et al., 2023), yet there is little to no research into how increases in dispositional mindfulness through training may affect these relationships and potential flow-on effects to symptomology.

Differentiating these subtypes may explain the conflicting results around the adaptivity of mind wandering, where the deliberate subtype (MWD) is primarily correlated with adaptive outcomes such as creativity (Agnoli et al., 2018; Carciofo & Jiang, 2021; Robison & Unsworth, 2018) and the spontaneous counterpart more strongly correlated maladaptive outcomes such as anxiety, depression, stress (Seli et al., 2019) and ADHD (Seli et al., 2015b). Global self-report measures of trait mind wandering, such as the Mind-Wandering Questionnaire (Mrazek et al., 2013), have been found to primarily measure MWS (Vannucci & Chiorri, 2018) which would bias results of mind wandering toward maladaptive outcomes. Much of the literature still measures mind wandering on a global scale rather than considering the subtypes.

### 1.2. Hypotheses

This study aimed to investigate an augmented version of the S-REF model as a potential mechanism of MBI symptomology-reduction effects to answer the following research questions: (1) What is the relationship between dispositional mindfulness and metacognitive beliefs, and (2) How do the subtypes of mind-wandering and rumination differ in their relationship with metacognitive beliefs and symptomology?

To investigate these questions, a randomised control trial of a MBI would be the most effective. However, these trials are expensive and time-consuming and only include a relatively small number of participants. This exploratory study was conducted to determine the likelihood of this augmented S-REF model being a mechanism of symptomology-reduction effects. It is hoped that in the future, RCTs may establish this more conclusively.

To this end, the relationships between mindfulness, metacognitive beliefs, the rumination and worry elements of the CAS, and symptomology of anxiety and depression were examined to make inferences about possible pathways between increases in mindfulness and decreases in symptomology often found in MBI research. The CAS was augmented to include subtypes of rumination and mind wandering, which are considered adaptive and maladaptive and related to mindfulness in prior research.

Given that meditation frequency is more predictive of symptomology than dispositional mindfulness (Lantheaume et al., 2024; Querstret et al., 2020), the between-group differences in DV scores and correlations between those scores were compared for participants self-reporting meditation frequency as daily, weekly or rarely. Self-reported meditation frequency was used to measure participant familiarity with mindfulness (rarely meditating = low familiarity with mindfulness; weekly meditating = some familiarity with mindfulness; daily meditating = good familiarity with mindfulness). While participants in this study did not undergo an MBI, it was thought that the rarely, weekly and daily meditating groups would approximate the dispositional mindfulness of individuals who had not attended an MBI, were amid an MBI and at the conclusion of an MBI, respectively, presuming the MBI involved formal meditation training.

It was expected that:

1. Mindfulness (FFMQ-15) would be inversely correlated with metacognitive beliefs (MCQ-30).
2. Metacognitive beliefs (MCQ-30) significantly associated with:
  - Brooding rumination (RRS-B) but not the reflective counterpart (RRS-R).
  - Spontaneous mind wandering (MWS) but not the deliberate (MWD) subtypes.
3. Brooding rumination (RRS-B) and spontaneous mind wandering (MWS) would be significantly correlated with symptomology of anxiety (GAD-7) and depression (PHQ-9), while the reflective (RRS-R) and deliberate (MWD) subtypes would not.
4. Daily meditators would be significantly higher in mindfulness and lower in metacognitive beliefs, brooding rumination, spontaneous mind wandering and symptomology of anxiety and depression than the weekly and rarely groups.

## 2. Materials and Methods

### 2.1. Study Design

The study was an exploratory study examining the relationships of dispositional mindfulness and meditation frequency with anxiety and depression symptomology, mind wandering, rumination, worry, and metacognitive beliefs. Participants were recruited through professional networks and snowball sampling (Tenzek, 2017) by way of email and social media. After giving consent, participants completed a battery of questionnaires online in one sitting using Qualtrics (https://www.qualtrics.com (accessed on 21 February 2021)), and only the data from completed questionnaires were recorded for analysis.

### 2.2. Participants

Complete data were recorded for *n* = 178 participants (*M_age_* = 53.13, *SD* = 11.8); see Table 1.

### 2.3. Measures

#### 2.3.1. Mindfulness

The 15-item version (FFMQ-15; Baer et al., 2012) of the Five Facets of Mindfulness Questionnaire (FFMQ; Baer et al., 2006) was used to measure dispositional mindfulness within five facets of observing, describing, acting with awareness, non-judging and non-reactivity. These facets are believed to comprise dispositional mindfulness in which one can consistently notice and attend to sensory and mental phenomena (observing), attach a semantic label to those experiences (describing), bring conscious awareness to one’s activities (acting with awareness), refrain from making judgements about cognitive and sensory experience (non-judging), and refrain from automatically responding to cognitive and sensory experience (non-reactivity). The instrument has good internal consistency and is used in various populations, with alphas ranging from 0.75 to 0.91 (Christopher et al., 2012).

#### 2.3.2. Rumination

The Ruminative Responses Scale (RRS; Treynor et al., 2003) comprises two five-item subscales measuring tendencies to engage in brooding or reflective rumination in response to stress. These items are scored on a scale that ranges from almost never (1) to almost always (4). Evaluations of these two factors have confirmed the instrument’s internal consistency for both the reflective (*α* = 0.79) and brooding (*α* = 0.79) subscales, in addition to test–retest reliability (*r* = 0.60 and *r* = 0.62, respectively) (Treynor et al., 2003).

#### 2.3.3. Mind Wandering

The Mind Wandering: Deliberate (MW-D) and Mind Wandering: Spontaneous (MW-S) scales (Carriere et al., 2013) are four-item self-report scales capturing individuals’ tendency to engage in adaptive or maladaptive mind wandering. The former (MW-D) has shown good psychometric properties (*α* = 0.88) and includes items such as: “I allow my thoughts to wander on purpose”. The latter (MW-S) also shows consistent psychometric properties (*α* = 0.90) (Carriere et al., 2013), including items such as “I find my thoughts wandering spontaneously” scored using a seven-point Likert scale.

#### 2.3.4. Worry

The Penn State Worry Questionnaire-A (PSWQA; Crittendon & Hopko, 2006) is an abbreviated form of the Penn State Worry Questionnaire (PSWQ; Meyer et al., 1990) that measures trait worry by way of an 8-item questionnaire consisting of 5-point Likert scales, ranging from “Not at all typical of me (1)” through to “Very typical of me (5)”. The measure has shown good internal consistency in older (*α* = 0.89) and younger (*α* = 0.94) adult populations, with excellent test–retest reliability for older (*r* = 0.92) and younger (*r* = 0.87) adults (Crittendon & Hopko, 2006).

#### 2.3.5. Metacognitive Beliefs

The Metacognitive Questionnaire (MCQ-30; Wells & Cartwright-Hatton, 2004) is a 30-item self-report questionnaire that measures metacognitive beliefs, consisting of five subcategories: positive beliefs about worry; negative beliefs about the uncontrollability and danger of thoughts and worry; cognitive confidence; beliefs about the need to control thoughts; and cognitive self-consciousness, rated on a 4-point Likert scale. Higher scores in each subcategory indicate a greater tendency toward pathological metacognitive activity and maladaptive metacognitive beliefs. The internal consistency of the MCQ-30 is excellent (α = 0.93), with the subscales ranging from 0.72 to 0.93, and the test–retest reliability is acceptable, with *r* ranging from 0.59 to 0.87 for the subscales (Wells & Cartwright-Hatton, 2004).

#### 2.3.6. Symptomology

The General Anxiety Disorder-7 (GAD-7; Spitzer et al., 2006) consists of 7 items, scored on a Likert scale from 0 (Not at all sure) to 3 (Nearly every day). It has shown excellent internal consistency (α = 0.92) and good reliability in test–retest (*r* = 0.83) (Spitzer et al., 2006).

The 9 items of the Patient Health Questionnaire-9 (Kroenke et al., 2001) are based on depression diagnostic criteria of the DSM-IV, scored on a Likert scale from 0 (not at all) to 3 (nearly every day). As a diagnostic tool to monitor depression severity and treatment, the measure is reliable (α = 0.89) (Kroenke et al., 2001) and well-supported across ethnicities, with α ranging between 0.79 and 0.86 (Huang et al., 2006).

#### 2.3.7. Meditation Frequency

Participants who reported having learned to meditate were then asked to report the frequency of their meditation practice (daily, weekly, rarely), with the expectation that regular meditators would be significantly higher than non-meditators in trait mindfulness as found in prior research (Lachaud et al., 2022). It was thought that between-group differences in scores and correlations between variables would give some insight into possible pathways of MBI effects by the groups reflecting changes that may occur in individuals throughout an MBI. That is, the mindfulness scores of the rarely group would approximate people at the beginning of an MBI, the weekly group as intermediate meditators, and daily as those who have completed an MBI and are regularly practising.

### 2.4. Statistical Analysis

All statistical analyses were conducted using the Statistical Package for the Social Sciences (SPSS; Version 26) program. Results were considered significant at *p* < 0.05. Assumptions were tested. Bonferroni corrections were applied as multiple comparisons were performed to adjust for Type I errors. An estimate of the required sample size was conducted a priori using G*Power (v3.1.9.4, Faul et al., 2007) with *α* = 0.05, *β* = 0.95, and between-groups ANOVA analysis for 3 groups required a sample size of *n* = 177 for a medium effect size of 0.3, and *t*-tests for independent means (one-tailed) required a sample size of *n* = 176 for a medium effect size of 0.5. One hundred and seventy-eight participants completed the survey; however, *n* = 18 reported never meditating, providing between-group analysis for frequency of meditation for *n* = 160. Significance would require greater effect sizes for *n* = 160, e.g., ANOVA of 0.32 and *t*-test of 0.53. These data were further analysed in relation to self-reported frequency of meditation practice, as this has been found to be more predictive of symptomology than mindfulness (Lantheaume et al., 2024; Querstret et al., 2020).

### 2.5. Human Ethics Approval

Human ethics approval was given by the UniSC Human Ethics Committee prior to the recruitment of participants; approval S201454.

## 3. Results

The internal consistency of each measure was tested, and Cronbach’s alpha is reported in Table 2.

A Shapiro–Wilk test for normality (*p* < 0.05) was not satisfied for any of the dependent variables. Thus, non-parametric results are reported here.

Spearman’s rank-order correlations were run to assess the relationship between variables, and the results are reported in Table 3.

A Williams’ test was conducted to compare two dependent correlations: the correlation between MWS and PHQ (*r* = 0.45) and the correlation between MWD and PHQ (*r* = 0.18), which share a common variable (PHQ), with a sample size of 160. The analysis revealed a statistically significant difference between the correlations, *t*(157) = 3.18, *p* = 0.002, indicating that MWS is more strongly associated with PHQ than MWD.

A Williams’ test was conducted to compare the dependent correlations between MWS and GAD-7 (*r* = 0.39) and MWD and GAD-7 (*r* = 0.20), with a shared variable (GAD-7) and a sample size of 160. The difference between the correlations was statistically significant, *t*(157) = 2.18, *p* = 0.031, suggesting that MWS is more strongly associated with GAD-7 than MWD.

A Williams’ test was conducted to compare the dependent correlations between RRS-B and PHQ (*r* = 0.60) and RRS-R and PHQ (*r* = 0.43), with PHQ as the shared variable. With a sample size of 160, the difference between the correlations was statistically significant, *t*(157) = 2.52, *p* = 0.013, indicating a stronger association between RRS-B and PHQ than RRS-R and PHQ.

A Williams’ test was conducted to compare the dependent correlations between RRS-B and GAD-7 (*r* = 0.58) and RRS-R and GAD-7 (*r* = 0.35), with GAD-7 as the shared variable. The analysis, based on a sample of 160 participants, revealed a statistically significant difference, *t*(157) = 3.27, *p* = 0.001, indicating that RRS-B is more strongly associated with GAD-7 than RRS-R.

Data underwent non-parametric Kruskal–Wallis H independent samples testing to determine differences in each dependent variable for the three frequency of meditation groups: daily (*n* = 73), weekly (*n* = 47), and rarely (*n* = 40), Table 4. The main effect for frequency was found for the *observing*, *acting with awareness* and *non-reactivity* facets of mindfulness, GAD-7 (anxiety), PHQ-9 (depression), MWD (deliberate mind wandering), RRS-B (brooding rumination), PSWQ (worry), and two subscales of the MCQ-30 (*cognitive confidence* and *positive beliefs about worry*). Although main effects were not found for all variables, pairwise comparisons were examined for any significant difference between groups in accordance with (Hsu, 1996, p. 177). The daily group scores were significantly higher than the rarely group on *observing*, *acting with awareness, and non-reactivity* facets of mindfulness and significantly lower for GAD-7 (anxiety), PHQ-9 (depression), MWD (deliberate mind wandering), PSWQ (worry), and the *positive beliefs about worry* subscale of the MCQ-30 (metacognitive beliefs).

ANCOVAs were used to determine the effect of meditation frequency (daily, weekly, rarely) on dependent variables after controlling the effect of the observing facet of mindfulness. There were no statistically significant differences in adjusted group means for meditation frequency for many of the dependent variables, except in the cases of *non-judging*, GAD-7, PHQ-9, MCQ-30 and *need to control.* The interpretability of the adjusted group comparisons for these cases is limited, and caution is warranted when drawing conclusions from these results (Table 5).

## 4. Discussion

The main purpose of this research was to investigate the S-REF model (Wells & Matthews, 1996, 2015) for possible pathways of MBI effect on anxiety and depression symptomology. The rumination element of the cognitive attentional syndrome (CAS) model was expanded to include brooding and reflective subtypes, while deliberate and spontaneous subtypes of mind wandering not currently considered part of the CAS were also included. Relationships between mindfulness, metacognitive beliefs, elements of the CAS, and symptomology of anxiety and depression were examined to determine potential pathways of effect through changes in dispositional mindfulness that are expected to occur through MBI training.

### 4.1. Mindfulness and Symptomology

The correlations reported in Table 2 revealed significant negative correlations of anxiety (GAD-7) and depression (PHQ-9) symptomology with all five facets of mindfulness (*observing*, *describing*, *acting with awareness*, *non-judging* and *non-reactivity*), which was as expected and supportive of prior research (Galante et al., 2021; B. T. Johnson et al., 2023; Medvedev et al., 2018). Those reporting to meditate daily also scored significantly higher on the *observing*, *acting with awareness* and *non-reactivity* facets of mindfulness (FFMQ-15) and significantly lower on anxiety (GAD-7) and depression (PHQ-9) than those who reported they rarely meditate. In this cohort, rarely meditators are those who have received meditation instruction at some point but meditate less than once a week. It was assumed that the dispositional mindfulness level for those who only meditate rarely would be similar to a baseline score for a non-meditator or a novice pre-MBI, while a daily meditator could approximate a participant post-MBI.

These direct correlations between mindfulness facets and symptomology of anxiety and depression indicate that some of the symptomology-reduction effects of MBIs likely occur as a direct result of mindfulness increases or through mechanisms beyond the scope of this research.

### 4.2. Mindfulness and Metacognitive Beliefs

For the S-REF model to be a candidate for MBI effects on symptomology, it must be possible for increases in dispositional mindfulness to affect the metacognitive beliefs posited as a driver of symptomology. This research found significant negative correlations of all five facets of mindfulness (FFMQ-15) the metacognitive beliefs questionnaire (MCQ-30), indicating that increases in mindfulness skills widely found to occur in MBIs are likely to play a role in decreasing these. Of the MCQ-30 subscales, *negative beliefs about danger and uncontrollability of worry* were significantly inversely correlated with all five facets of mindfulness. In comparison, the *need to control thoughts* subscale was significantly correlated with four of the five facets (*observing, describing, non-judging and non-reactivity*). Based on prior research that found these aspects of metacognitive beliefs to be an important part of anxiety and depression disorders (S. U. Johnson & Hoffart, 2018; Wells, 2010), these results indicate that increases in dispositional mindfulness occurring through an MBI could instigate decreases in these metacognitive beliefs, initiating a mechanism of symptomology reductions through the effects on the CAS.

In explicitly targeting these beliefs, metacognitive therapy incorporates a detached mindfulness component that differs from popularised mindfulness approaches (Wells, 2005a). The significant negative correlations of mindfulness facets with metacognitive beliefs (MCQ-30) and many of its subscales in this study suggest that these beliefs may also be affected by mindfulness training approaches extant in more commonly used MBIs despite not being an explicit target of these interventions. Perhaps formal mindfulness training that increases an ability to remain focused on a particular object (often the breath) without being distracted by other cognitions competing for that attention gives an implicit and experiential knowledge that undermines the veracity of these beliefs. Significantly higher scores on the *observing*, *acting with awareness*, and *non-reactivity* facets of mindfulness for those reporting to meditate daily versus rarely further suggest that regular meditation may have a protective role against psychopathologies through this significant inverse relationship with metacognitive beliefs.

### 4.3. Metacognitive Beliefs and the CAS

#### 4.3.1. Worry-Symptomology Pathway

Metacognitive beliefs (MCQ-30) and four of its five subscales showed strong (*cognitive confidence, positive beliefs about worry and negative beliefs*) and moderate (*need to control thoughts*) positive significant correlations with worry, which was in turn very strongly and positively correlated with both anxiety and depression symptomology. This was expected, as these relationships are an important part of the S-REF model. When considered in relation to the significant negative correlation of mindfulness and metacognitive beliefs, these relationships reveal a potential pathway of MBI-driven symptomology reduction in which increases in dispositional mindfulness may result in reduced maladaptive metacognitive beliefs, thereby reducing the likelihood of type 2 worry-instigating symptomology. While the correlational results of this study cannot make inferences on the directionality of the relationship between mindfulness and metacognitive beliefs, this pathway fits neatly with the S-REF model and prior research that found those beliefs to have a causal relationship with symptomology (Capobianco et al., 2019).

Of the MCQ-30 and its subscales, the only significant difference between daily and rarely meditators was on the *positive beliefs about worry* subscale of the MCQ-30. While this was somewhat of a surprise, lower scores on this subscale are associated with individuals being less prone to consider worry an effective coping strategy to deal with stressors and are likely to have played a key role in the daily groups’ significantly lower worry (PSWQ) scores than the rarely group. These lower scores could make the daily meditators less likely to succumb to type 2 meta-worry, thereby reducing, or perhaps even avoiding, a potential pathway to symptomology.

It is worth noting that all five facets of mindfulness were found to have a significant negative correlation with worry (PSWQ), which would suggest that mindfulness impacts worry directly and/or through pathways other than the S-REF model investigated in this study.

#### 4.3.2. Rumination-Symptomology Pathway

While prior research has found links between metacognitive beliefs and rumination (Hallard et al., 2021; Papageorgiou & Wells, 2003), more research is needed into the effect of mindfulness on the reflective and brooding subtypes. To the best of our knowledge, this is the only study to have investigated these subtypes as part of the cognitive attentional syndrome (CAS).

The different relationships reflective and brooding rumination were found to have with other variables support prior research that found these subtypes to be distinguishable despite their significant positive correlation (Olatunji et al., 2013). Brooding rumination was found to have significant positive correlations with metacognitive beliefs (MCQ-30), four of its five subscales (*cognitive confidence, positive beliefs about worry, negative beliefs about uncontrollability and danger, and need to control thoughts*) and symptomology of anxiety and depression, all of which is consistent with the S-REF model, in which rumination is considered an important element of the CAS. The reflective subtype was also correlated significantly with metacognitive beliefs, and four of the five subscales (*positive beliefs about worry, cognitive self-consciousness, negative beliefs about uncontrollability and danger, and the need to control thoughts*) were weaker than the brooding counterpart. This pattern of weaker associations for the reflective subtype is similar to prior research that found weaker relationships for reflective compared to brooding rumination in significant correlations with depression symptomology (Grassia & Gibb, 2008; Trew & Alden, 2009), worry and anxiety (Olatunji et al., 2013). While these data are suggestive of reflective rumination being less related to negative outcomes, these significant positive correlations do cast doubt on some characterisation of the reflective subtype as an adaptive form of rumination (Schoofs et al., 2010; Treynor et al., 2003).

As a potential pathway of MBI effects on symptomology, the reduction in metacognitive beliefs through mindfulness training could flow onto lower rumination, particularly the brooding subtype, which may lead to reduced anxiety and depression symptomology. As far as we know, there is no research into relationships between these metacognitive beliefs and rumination subtypes. However, it is plausible that lower metacognitive beliefs might mediate a tendency to engage in reflective rumination more than brooding. Although these results implicate reflective rumination as a weak pathway to symptomology, a shift in tendency away from brooding to reflective rumination through MBI training may still result in reduced symptomology scores through weaker associations with metacognitive beliefs and symptomology.

Brooding rumination was also found to have a significant negative correlation with all five facets of mindfulness. In contrast, reflective rumination was found to have a significant but weaker negative correlation with only the *non-judging* and *non-reactivity* facets. Similar variance in the relationship between mindfulness and rumination subtypes has been found previously, implying direct effects of mindfulness on rumination subtypes that do not depend on reductions in metacognitive beliefs.

#### 4.3.3. Mind Wandering–Symptomology Pathway

Significant positive correlation of the spontaneous (MWS) subtype with metacognitive beliefs (MCQ-30), three of its subscales (*cognitive confidence*, *positive beliefs about worry* and *negative beliefs about the danger and uncontrollability of worry*)*,* worry (PSWQ)*,* and symptomology of anxiety (GAD-7) and depression (PHQ-9) flag it as an important part of the conversation about pathways of MBI effects. Conversely, the only significant correlation the deliberate mind wandering (MWD) subtype was found to have with metacognitive beliefs was with the *positive beliefs about the worry* subscale of the MCQ, and its significant correlations with anxiety and depression symptomology were both weaker than the spontaneous counterpart. In this pathway model, reductions in metacognitive beliefs through increases in dispositional mindfulness achieved by MBI participation could reduce spontaneous mind wandering and its flow-on effects on anxiety and depression symptomology. Evidence of this pathway as part of a transdiagnostic model can find some support in prior research that found intentional mind wandering to have a significantly weaker relationship than its spontaneous counterpart with anxiety and depression (Seli et al., 2019). Similarly, significant correlations have been found for spontaneous mind wandering (MWS) but not for the deliberate (MWD) subtype with attention deficit hyperactivity disorder (ADHD) (Seli et al., 2015b) and obsessive–compulsive disorder (OCD) (Cole & Tubbs, 2022).

The significant correlations of spontaneous mind wandering (MWS) with all but the *describing* facet of mindfulness (FFMQ-15), while deliberate mind wandering was significantly correlated with only one (*acting with awareness*) facet, are similar to past results (Seli et al., 2015a) which found the only significant correlation of deliberate mind-wandering (MWD) to be with *non-reactivity*, while the spontaneous subtype (MWS) was significantly correlated with all five mindfulness FFMQ facets (*observing*, *describing*, *acting with awareness*, *non-judging,* and *non-reactivity*). A lack of any significant positive correlations between mindfulness and the deliberate subtype (MWD) is one noticeable difference between this and the results of Seli et al. (2015a). However, the pattern of consistently weaker associations for deliberate mind wandering (MWD) than spontaneous mind wandering (MWS) is the same in both studies. Differences seen here may be due to the high prevalence of meditation experience among participants of this study. These results point to mindfulness facets affecting spontaneous mind wandering directly and by way of mechanisms not captured in the current research.

### 4.4. Mind Wandering and Rumination as Continua or Categorical

Prior research has found mind wandering to be associated with worry and metacognitive beliefs (Carciofo et al., 2017). However, this research is the first indication of the difference in the relationship between these beliefs and subtypes of mind wandering and rumination. The significant positive inter-correlations of mind wandering (MWD–MWS) and rumination (RRS-R–RRS-B) subtypes point to some overlap in each of these constructs, while the consistent pattern of significantly weaker correlations of deliberate mind wandering (MWD) and reflective rumination (RRS-R) to other DVs than their corresponding spontaneous (MWS) and brooding (RRS-B) subtypes supports prior research that characterised them as related and distinguishable (Nolen-Hoeksema et al., 2008; Seli et al., 2015a). This raises a subtle but important possibility that mind wandering and rumination subtypes might be more accurately represented as extremes on their respective continua rather than the current categorical model of distinct typologies measured independently of each other.

For example, although the content of a mind-wandering episode might be the same in two different instances, the individual’s intention to engage with that content may determine whether it could be considered deliberate (MWD) or spontaneous (MWS). Likewise, for rumination, it may not be the content of a rumination episode but the degree of problem-solving (Bernblum & Mor, 2010; Treynor et al., 2003) that plays an important role in determining where it lies on a continuum between reflective (RRS-R) and brooding (RRS-B). In this adjusted model of continua of adaptability, increased mindfulness achieved through MBI training may flow on to reductions in metacognitive beliefs, thereby increasing an individual’s proclivity to relate to the content of their mind-wandering and rumination in more adaptive ways. This tendency toward more adaptive treatment of cognition would render the content of mind wandering and rumination episodes increasingly inert, resulting in a reduction in current symptomology and future risk of anxiety and depression. This reconceptualisation of subtypes as continuous variables would align them with many other psychometric measures such as metacognitive beliefs, worry, and symptomology measured on continua of adaptivity.

This proposed model of rumination and mind-wandering supports, expands and adds nuance to the assertions of (Nolen-Hoeksema, 1991; Teasdale, 1988) that an individual’s relationship to their mental events may be more important than the content of those events. The current research indicates that mindfulness training may mediate these relationships by shifting individuals’ tendency to engage in maladaptive mind-wandering and/or rumination to their more adaptive subtypes. In doing so, this reveals a potential pathway of mindfulness effects on symptomology and a pathway by which these symptomologies become amplified. This understanding could help adapt current treatments and MBIs to be more effective in symptomology reduction.

In short, the pattern of relationship between mind wandering and rumination subtypes with anxiety, depression, metacognitive beliefs, worry, and mindfulness gives cause to investigate the possibility of mind wandering and rumination subtypes as existing on continua rather than the current categorical model. If accurate, this model could offer a deeper explanation of the current relationship between these phenomena and reveal possible pathways of MBI effects on symptomology and the cognitive attentional syndrome (CAS) while also explaining some apparent contradictory findings on the adaptability of mind wandering (Agnoli et al., 2018; Carciofo et al., 2017) when treated as a single, global construct.

#### Dereification as a Fundamental Mechanism

Significant negative correlations of mindfulness facets (FFMQ-15) with spontaneous mind wandering (MWS) and brooding rumination (RRS-B) are indicative of a direct link or alternative mechanisms of MBI symptomology reduction not captured in this research. One possible candidate for this relationship is the dereification dimension in the model of Lutz et al. (2015), which is defined as the extent to which cognitions and feelings are distinguished as subjective processes instead of infallible representations of reality. In this model, increased dereification is considered an aim of all mindfulness practices and may be an underlying mechanism of MBI effects influencing not just symptomology, mind wandering and cognitive attentional syndrome but the metacognitions themselves. This line of research can already be found in relation to psychological concepts such as cognitive fusion (Gillanders et al., 2014) and decentering (Williams, 2010), both of which are related (but not identical) to dereification (Lutz et al., 2015), and may explain some of the correlations between mindfulness, the CAS and symptomology that do not rely on metacognitive beliefs.

### 4.5. Limitations

The high level of exposure to mindfulness training in this cohort may not reflect responses of a more general population, and neither the overall lifetime experience nor type of practice have been taken into account in this study. Different types of mindfulness and meditation training can be expected to yield different effects, yet this study included all types of participant meditation training in a coarse operationalisation of mindfulness, from which conclusions must be drawn with some caution.

It should be noted that response shift in which interpretations of questionnaire items can differ between novice and more experienced meditators is an issue raised in prior reviews of mindfulness literature (Grossman, 2008; Van Dam et al., 2018), which means any cross-study comparisons need to be carried out with some caution. There is some evidence that the observing facet of the FFMQ is interpreted differently by experienced meditators compared to novices (Baer et al., 2006; Gu et al., 2016), which would also confound the comparison of daily and rarely meditators of this study and results of prior research. In this study, the adjusted group means for meditation frequency were significantly different for *non-judging*, GAD-7, PHQ-9, and MCQ-30 and *needed to be controlled*, limiting the interpretability of the adjusted group comparisons in these cases.

Furthermore, the directionality of these relationships is not explored in the correlational analysis.

## 5. Conclusions

This research examined the S-REF model of emotional disorder as a possible mechanism of MBI symptom reduction effects. Its goal was to better understand the mechanisms driving these effects, improve the efficacy of MBIs, and inform future treatment and prevention.

Results revealed a likelihood that increases in mindfulness through MBI training could decrease metacognitive beliefs, initiating decreases in the rumination and worry elements of the CAS and spontaneous mind wandering, which could be expected to play a role in reduced symptomology scores over the duration of MBI. These results are congruent with those of O’Toole et al. (2019), who established a temporal sequence of increased metacognitive abilities and GAD symptomology reduction. Additional significant negative correlations between mindfulness, the CAS elements, mind wandering, and symptomology indicate potential MBI effect pathways that do not involve metacognitive beliefs. Dereification is posited as a potential explanation for these correlations and would likely play a role in any reduction in metacognitive beliefs through MBI training.

Those with a daily meditation practice were found to have significantly higher trait mindfulness, which appears to have a protective effect on the CAS and symptomology.

Evidence was also found to justify investigating a re-conceptualising of mind wandering and rumination subtypes as existing at either end of their respective continua rather than the current categorical model. This subtle change in approach may resolve some debate about the adaptability of mind wandering and rumination, perhaps even leading to a clearer understanding of some currently opaque mechanisms of MBI effects.

This research found evidence of an augmented S-REF model as a likely mechanism of mindfulness symptomology-reduction effects. In doing so, it indicates that increases in mindfulness may alter a subject’s relationship with the content of maladaptive cognitive processes in such a way that the pathway to anxiety and depression symptomology is reduced or perhaps severed, which could open the way for more effective MBIs by explicitly targeting these mechanisms for change. Additionally, other treatments that target these mechanisms could be developed for populations unable or unwilling to engage in mindfulness training. In either case, the efficacy of non-pharmacological treatments and treatment adjuncts could be developed and refined to alleviate anxiety and depression. The transdiagnostic nature of these mechanisms may also make them suitable for successful treatment of other psychopathologies.

### Future Research Direction

An important next step would be to measure changes in mindfulness, metacognitive beliefs, the CAS, and symptomology over the duration of an MBI. Given the variety of MBIs and the prevalence of ancillary factors such as journaling, yoga, and psychoeducation in MBI protocols, an ancillary-free MBI focused exclusively on mindfulness training would offer the clearest understanding of how changes in these measures impact symptomology.

The possible role of dereification in determining the difference between mind wandering and rumination subtypes should be further explored. This could be supported by examining whether the subtypes are better represented as extremes of continua or per the current categorical model.

While this research investigated effects on anxiety and depression symptomology, the transdiagnostic nature of the S-REF model means that this pathway may be effective in a wider variety of symptoms. This could be especially relevant for disorders such as Post-Traumatic Stress Disorder (PTSD) and ADHD, where the power of memories and other cognitive events to induce maladaptive responding might be reduced through MBI-driven dereification effects.

Finally, the continuous or categorical nature of mind wandering and rumination subtypes should be clarified to better understand their relationship with mindfulness, their role in the S-REF model, and their direct effects on symptomology.

## Figures and Tables

**Table 1:** Sociodemographic characteristics of participants at baseline.

Characteristic	*n*	%
Gender		
Female	143	80.33
Male	34	19.10
Prefer not to say	1	0.56
Marital status		
Married	86	48.31
Divorced/widowed/separated	41	23.03
Never married	51	28.65
Children		
0	70	39.33
1	35	19.66
2	45	25.28
3+	29	16.29
Highest educational level		
Vocational training or less	49	27.53
Undergraduate degree	48	26.97
Postgraduate degree	81	45.51
Mental health diagnosis		
Yes	68	38.2
No	110	61.80

Note. *n* = 178 Participants were, on average, 53.14 years old (*SD* = 11.76).

**Table 2:** Mean, SD, and Cronbach’s alpha values for variables.

	*M* (*SD*) *n* = 178	Range	Cronbach Alpha	Skew	Kurtosis
Observing	10.48 (2.20)	3–15	0.66	−0.28	0.20
Describing	12.76 (2.60)	3–15	0.86	−0.56	−0.08
Act Aware	15.46 (2.17)	3–15	0.79	0.22	0.16
Non-judging	16.95 (2.78)	3–15	0.86	−0.31	−0.36
Non-reactivity	9.86 (2.60)	3–15	0.87	0.06	−0.36
GAD-7	6.19 (4.97)	0–21	0.90	0.90	0.15
PHQ-9	6.51 (5.73)	0–27	0.90	1.13	0.65
MWD	12.65 (5.64)	4–28	0.84	0.35	−0.54
MWS	15.46 (5.93)	4–28	0.87	−0.01	−0.65
PSWQ	22.37 (9.17)	8–40	0.95	0.04	−1.01
RRS-R	10.73 (3.54)	5–20	0.81	0.24	−0.51
RRS-B	10.31 (3.49)	5–20	0.84	0.65	0.42
MCQ-30	59.89 (12.82)	30–120	0.88	−0.16	1.17
Cognitive confidence	11.43 (4.18)	6–24	0.88	0.75	−0.04
Positive Beliefs	9.59 (3.84)	6–24	0.90	0.99	0.23
Cognitive Self-consciousness	16.51 (4.19)	6–24	0.84	−0.18	−0.71
Negative Beliefs	12.49 (4.82)	6–24	0.86	0.53	−0.64
Need to control Thoughts	9.87 (3.26)	6–24	0.71	0.93	0.42

**Table 3:** Spearman correlations coefficient of variables.

	1. Observing	2. Describing	3. Acting W Awareness	4. Non-Reactive	5. Non-Judging	6. GAD-7	7. PHQ-9	8. MWD	9. MWS	10. RRS-R	11. RRS-B	12. PSWQ	13. MCQ-30	14. Cognitive Confidence	15. Positive Beliefs	16. Cognitive Self-Conscious	17. Negative Beliefs	18. Need to Control
1	-																	
2	0.27 **	-																
3	0.39 **	0.21 **	-															
4	0.35 **	0.37 **	0.38 **	-														
5	0.39 **	0.27 **	0.43 **	0.48 **	-													
6	−0.27 **	−0.29 **	−0.37 **	−0.51 **	−0.53 **	-												
7	−0.25 **	−0.34 **	−0.31 **	−0.53 **	−0.56 **	0.80 **	-											
8	0.07	−0.08	−0.18 *	−0.07	−0.08	0.20 **	0.18 *	-										
9	−0.22 **	−0.08	−0.47 **	−0.27 **	−0.37 **	0.39 **	0.45 **	0.29 **	-									
10	0.16 *	−0.11	−0.12	−0.19 *	−0.17 *	0.35 **	0.43 **	0.28 **	0.27 **	-								
11	−0.25 **	−0.20 **	−0.27 **	−0.49 **	−0.51 **	0.58 **	0.60 **	0.10	0.35 **	0.40 **	-							
12	−0.26 **	−0.27 **	−0.41 **	−0.52 **	−0.62 **	0.74 **	0.72 **	0.18 *	0.49 **	0.30 **	0.66 **	-						
13	−0.21 **	−0.25 **	−0.31 **	−0.54 **	−0.40 **	0.57 **	0.58 **	0.11	0.39 **	0.40 **	0.59 **	0.67 **	-					
14	−0.31 **	−0.27 **	−0.35 **	−0.35 **	−0.29 **	0.32 **	0.36 **	0.00	0.37 **	0.11	0.34 **	0.40 **	0.59 **	-				
15	−0.22 **	−0.10	−0.27 **	−0.26 **	−0.26 **	0.40 **	0.32 **	0.25 **	0.20 **	0.27 **	0.36 **	0.49 **	0.62 **	0.25 **	-			
16	0.26 **	0.13	0.07	0.01	0.14	0.06	0.02	0.11	0.12	0.46 **	0.14	0.05	0.43 **	−0.03	0.11	-		
17	−0.25 **	−0.26 **	−0.35 **	−0.58 **	−0.55 **	0.62 **	0.63 **	0.02	0.40 **	0.29 **	0.60 **	0.72 **	0.77 **	0.37 **	0.35 **	0.11	-	
18	−0.18 *	−0.27 **	−0.09	−0.51 **	−0.22 **	0.32 **	0.41 **	0.02	0.07	0.21 **	0.39 **	0.36 **	0.71 **	0.33 **	0.38 **	0.14	0.52 **	-

* *p* < 0.05. ** *p* < 0.01.

**Table 4:** Descriptive statistics for the entire cohort divided into rarely, weekly and daily meditators, including significant differences between those groups, e.g., ‘rarely < weekly and daily’ indicates that the rarely group is significantly lower on DV compared to weekly and daily groups.

		Frequency of Meditation			Main and Significant Pairwise Results
	Total (*n* = 160) *M* (*SD*)	Rarely (*n* = 40) *M* (*SD*)	Weekly (*n* = 47) *M* (*SD*)	Daily (*n* = 73) *M* (*SD*)	Kruskal–Wallis H Independent Samples Test
Observing	10.49 (2.16)	9.48 (1.99)	10.96 (2.13)	10.75 (2.12)	*χ*^2^(2) = 14.01, *p* < 0.001 rarely < weekly and daily
Describing	12.78 (2.54)	12.28 (2.28)	12.45 (2.68)	13.27 (2.53)	*χ*^2^(2) = 5.73, *p* = 0.057
Act Aware	15.39 (2.19)	14.53 (2.09)	15.38 (1.97)	15.88 (2.26)	*χ*^2^(2) = 8.23, *p* = 0.016 rarely < daily
Non-judging	16.98 (2.84)	16.52 (2.96)	16.79 (2.55)	17.36 (2.94)	*χ*^2^(2) = 2.73, *p* = 0.255
Non-reactivity	9.84 (2.61)	8.95 (2.75)	9.91 (2.15)	10.27 (2.71)	*χ*^2^(2) = 7.75, *p* = 0.021 rarely < daily
GAD-7	6.21 (5.04)	8.05 (5.49)	5.85 (4.49)	5.44 (4.92)	*χ*^2^(2) = 7.61, *p* = 0.022 rarely > daily
PHQ-9	6.48 (5.76)	7.95 (5.61)	6.21 (5.12)	5.84 (6.15)	*χ*^2^(2) = 6.40, *p* = 0.041 rarely > daily
MWD	12.84 (5.73)	14.18 (5.96)	14.79 (5.22)	10.85 (5.32)	*χ*^2^(2) = 16.65, *p* < 0.001 rarely and weekly > daily
MWS	15.86 (5.74)	16.55 (5.65)	16.60 (4.84)	15.00 (6.26)	*χ*^2^(2) = 1.87, *p* = 0.392
PSWQ	22.43 (9.22)	25.78 (9.47)	23.34 (8.20)	20.00 (9.14)	*χ*^2^(2) = 11.15, *p* = 0.004 rarely > daily
RRS-R	10.98 (3.55)	11.23 (3.45)	11.28 (3.66)	10.66 (3.56)	*χ*^2^(2) = 1.06, *p* = 0.589
RRS-B	10.32 (3.54)	10.33 (3.08)	11.13 (3.33)	9.79 (3.84)	*χ*^2^(2) = 6.09, *p* = 0.048 rarely < daily
MCQ-30	60.41 (13.02)	63.10 (13.51)	62.02 (14.91)	57.90 (11.02)	*χ*^2^(2) = 3.72, *p* = 0.056
Cognitive confidence	11.53 (4.15)	12.3 (4.49)	12.43 (4.42)	10.53 (3.59)	*χ*^2^(2) = 6.87, *p* = 0.032
Positive Beliefs	9.60 (3.88)	11.05 (4.22)	9.91 (4.33)	8.60 (3.07)	*χ*^2^(2) = 8.59, *p* = 0.011 rarely > daily
Cognitive Self-consciousness	16.93 (4.14)	16.20 (3.79)	17.47 (3.96)	16.97 (4.43)	*χ*^2^(2) = 2.72, *p* = 0.257
Negative Beliefs	12.53 (4.87)	13.63 (4.97)	12.32 (5.02)	12.05 (4.69)	*χ*^2^(2) = 3.00, *p* = 0.224
Need to control Thoughts	9.83 (3.28)	9.93 (3.61)	9.89 (3.13)	9.74 (3.23)	*χ*^2^(2) = 0.12, *p* = 0.941

**Table 5:** ANCOVA results after adjusting for Observe facet scores across meditation frequency (daily, weekly, rarely).

	F	Sig.	Partial Eta Squared
Describe	0.72	0.487	0.01
Acting with Awareness	0.94	0.392	0.01
Non-judging	4.23	0.016	0.15
Non-reactivity	0.48	0.618	0.01
GAD-7	3.25	0.042	0.04
PHQ-9	4.51	0.013	0.06
MWD	2.63	0.075	0.03
MWS	0.45	0.637	0.01
PSWQ	1.94	0.147	0.03
RRS-B	0.84	0.434	0.01
RRS-R	0.29	0.748	0.00
MCQ-30	4.23	0.016	0.05
Cognitive Confidence	2.33	0.101	0.03
Positive Beliefs	0.43	0.650	0.01
Cognitive Self Confidence	2.44	0.091	0.03
Negative beliefs	2.01	0.138	0.03
Need to Control	3.90	0.022	0.05

## Data Availability

Data for this study is available upon request. Participants were informed that any future use of their data would be by the current research team.
